# Mapping Changes in Glutamate with Glutamate-Weighted MRI in Forced Swim Test Model of Depression in Rats

**DOI:** 10.3390/biomedicines12020384

**Published:** 2024-02-07

**Authors:** Donghoon Lee, Chul-Woong Woo, Hwon Heo, Yousun Ko, Ji Sung Jang, Seongwon Na, Nari Kim, Dong-Cheol Woo, Kyung Won Kim, Do-Wan Lee

**Affiliations:** 1 Faculty of Health Sciences, Higher Colleges of Technology, Fujairah P.O. Box 1626, United Arab Emirates; dlee@hct.ac.ae; 2 Convergence Medicine Research Center, Asan Institute for Life Sciences, Asan Medical Center, Seoul 05505, Republic of Korea; wandj79@hanmail.net (C.-W.W.); dcwoo@amc.seoul.kr (D.-C.W.); 3 Department of Convergence Medicine, University of Ulsan College of Medicine, Asan Medical Center, Seoul 05505, Republic of Korea; heohwon@gmail.com; 4 Department of Radiology, University of Ulsan College of Medicine, Asan Medical Center, Seoul 05505, Republic of Korea; ko.yousun82@gmail.com; 5 Biomedical Research Center, Asan Institute for Life Sciences, Asan Medical Center, Seoul 05505, Republic of Korea; etmira8787@gmail.com (J.S.J.); 87nasw@gmail.com (S.N.); 6 Department of Medical Science, Asan Medical Institute of Convergence Science and Technology, Asan Medical Center, University of Ulsan College of Medicine, Seoul 05505, Republic of Korea; nari.kim.0908@gmail.com

**Keywords:** forced swimming test, depression model, GluCEST, hippocampus, MR imaging

## Abstract

Chemical exchange saturation transfer with glutamate (GluCEST) imaging is a novel technique for the non-invasive detection and quantification of cerebral Glu levels in neuromolecular processes. Here we used GluCEST imaging and ^1^H magnetic resonance spectroscopy (^1^H MRS) to assess in vivo changes in Glu signals within the hippocampus in a rat model of depression induced by a forced swim test. The forced swimming test (FST) group exhibited markedly reduced GluCEST-weighted levels and Glu concentrations when examined using ^1^H MRS in the hippocampal region compared to the control group (GluCEST-weighted levels: 3.67 ± 0.81% vs. 5.02 ± 0.44%, *p* < 0.001; and Glu concentrations: 6.560 ± 0.292 μmol/g vs. 7.133 ± 0.397 μmol/g, *p* = 0.001). Our results indicate that GluCEST imaging is a distinctive approach to detecting and monitoring Glu levels in a rat model of depression. Furthermore, the application of GluCEST imaging may provide a deeper insight into the neurochemical involvement of glutamate in various psychiatric disorders.

## 1. Introduction

According to the diagnostic and statistical manual of mental disorders (DSM-5), major depressive disorder is characterized by identifiable alterations in mood, cognition, and neurovegetative functions, with episodes persisting for a minimum duration of two weeks [1]. The most representative symptoms of depression include sadness, emptiness, despair, sleep disturbances (e.g., insomnia), anxiety, agitation, restlessness, and suicidal ideations or actions, including suicide attempts [2,3]. Moreover, excessive or chronic stress can trigger an overproduction of cortisol, which is associated with various abnormal metabolic responses and neurodegenerative processes in the brain, potentially leading to conditions such as cortisol excess (e.g., Cushing’s syndrome) [4]. These phenomena may influence various regulatory functions of the brain, such as emotion regulation, memory, learning, and energy levels [5]. However, the diagnosis and treatment of depression rely on the relative and subjective evaluation of various symptoms representing diverse endophenotypes [6]. Owing to these diagnostic limitations, objectively assessing disease severity is challenging and treatment effects are inconsistent, rendering it a heterogeneous disease [6,7].

To date, numerous studies have aimed to uncover the underlying mechanisms and fine tune the diagnosis of depression, and research related to changes in brain metabolites and abnormal responses has been widely applied [8,9]. Previous studies reported the dysregulation of the primary excitatory neurotransmitter glutamate (Glu) in patients with major depressive disorder [10,11,12,13] and animal models [2,14,15,16,17]. Especially, the dysregulation of Glu in depressive disorders has been consistently linked to various pathological changes, encompassing the Glu–glutamine cycle, Glu excitotoxicity, hippocampal neurogenesis and function, stress response, and psychiatric health aspects, including mood regulation [18,19,20]. Hence, the identification of alterations in cerebral Glu levels in depressive disorders, along with visualizing and quantitatively evaluating specific regions of interest, may be crucial for observing mood regulation linked to depression [18,20].

Research interest in the hippocampal region in depressive disorders has been steadily increasing, as it is well recognized as an important area that can lead to various changes in brain function [21,22]. The non-invasive technique of in vivo ^1^H MRS, capable of exploring the neurochemical profile of living tissues involved in neurochemical processes, proves highly valuable in depression disorder research, particularly for sensitively observing metabolic changes within the hippocampal region [23]. The single voxel in ^1^H MRS is typically defined as a square or rectangular shape, which may impose limitations due to the potential inclusion of surrounding tissues within the voxel [24]. However, by optimizing the size and orientation of the target region, it is possible to sensitively detect in vivo metabolic signals within the hippocampal region, leveraging its high signal intensity [25]. Nevertheless, while ^1^H MRS exhibits ample sensitivity to detect neurotransmitters in different brain regions, it lacks the necessary spatial resolution for effective quantitative brain mapping and the visualization of target areas [26,27].

The chemical exchange saturation transfer (CEST) technique is a relatively recent advancement in MRI [26,28,29,30]. This method involves the application of a frequency-selective radiofrequency (RF) irradiation pulse to specific exchangeable protons (such as hydroxyls, amides, and amines) [31,32]. Consequently, this leads to diminished water signals, which can be quantified by a reduction in the water signal intensity. This indirect measurement allows the characterization of the solution microenvironment [27,33]. Glu-weighted CEST (GluCEST) imaging is a novel non-invasive in vivo technique used to analyze brain Glu distribution that offers significantly heightened sensitivity and superior spatial resolution compared to the conventional ^1^H-MRS approach [31]. The in vivo imaging of Glu levels using GluCEST was successfully demonstrated in a range of brain studies [27,28].

The forced swim test (FST) stands as one of the prominent methods for modeling depression in animal subjects and is commonly employed as an experimental model to assess depressive-like behavior [34]. During the FST, animals are subjected to involuntary placement in a water tank, and their floating or immobile state is monitored for a predetermined duration [35,36]. This protocol is postulated to elicit sentiments of helplessness and despair in animals, consequently resulting in the manifestation of behaviors commonly associated with depression [35,36]. One of the main assumptions of the FST modeling method is that this test imposes stress on animals, leading to changes in brain activity and various neurotransmitters [34,35]. Therefore, the depression modeling method utilizing the FST is considered suitable for assessing the impact of stress on cerebral metabolic phenomena [36,37,38].

Here we aimed to assess the in vivo changes in Glu signals that occur within the hippocampal region using GluCEST imaging and ^1^H MRS at 7 T in a rat model of depression induced by the forced swim test. We also conducted multi-parametric magnetic resonance imaging (MRI) using the same model to evaluate the potential factors influencing CEST signaling.

## 2. Materials and Methods

### 2.1. Animals

Animal care and experimental procedures were carried out in accordance with the approval granted by the Animal Care and Use Committee of Asan Medical Center, University of Ulsan College of Medicine (approval date: 29 January 2019; permit code: 2019-12-023).

Twenty-four rats were randomly assigned to either the FST group (*n* = 12) or the control group (*n* = 12). Each rat in the FST group was individually compelled to swim in a Plexiglas cylinder (measuring 60 cm in height and 25 cm in diameter) filled with water maintained at a temperature of 23–25 °C to a depth of 40 cm [39]. All rats in the FST group underwent two swimming sessions: an initial pre-swimming trial lasting 15 min, followed by a 10 min test conducted 24 h later [40]. Water replacement was carried out between each individual test to ensure consistency. After the swim test in the cylinder, they were removed and allowed to dry for 20 min in a heated enclosure (30 °C) before being returned to their individual cages. To minimize factors potentially influencing brain metabolism beyond the stress induced by forced swimming test, individual rest periods of approximately 30 to 40 min were provided to allow rats to return to their pre-swimming body temperature. After the rats’ body temperature had fully recovered to their pre-swimming level, the sequential acquisition of in vivo GluCEST/MRS and multi-parametric MR data was performed.

### 2.2. Data Collection

#### 2.2.1. MRI Scanner

The sequential acquisition of GluCEST, ^1^H-MRS, and multi-parametric MRI data was performed using a 7 T MR system (PharmaScan 70/16 scanner; Bruker BioSpin GmbH, Ettlingen, Germany) equipped with 400.0 mT/m self-shielding gradient system and an actively decoupled cross-coil setup. A 7.2 cm (inner diameter) RF volume coil was used for excitation, while a 2.5 cm single-loop surface coil was positioned atop each rats’ brain for signal reception. Each rat’s head was tightly adjusted using ear bars and a bite bar to minimize head movement.

#### 2.2.2. GluCEST Imaging

GluCEST imaging was conducted on a specifically chosen single slice with good visualization of hippocampus utilizing the following parameters: a fat-suppressed rapid acquisition with relaxation enhancement (RARE) sequence; slice thickness of 1.5 mm; field of view measuring 30 × 30 mm^2^; repetition time (TR) of 4.2 s; echo time (TE) of 36.4 ms; RARE factor set at 16; echo spacing of 6.1 ms; and a continuous-wave RF saturation pulse (power/length) of 3.6 μT/1000 ms. Z-spectra were obtained at 25 frequency offsets ranging from +6 to −6 ppm, with a step size of 0.5 ppm, and one unsaturated reference image (S_0_) was derived from the selected single-slice image [26,41].

To correct for B_0_ and B_1_ inhomogeneity, water saturation shift-referencing (WASSR) Z-spectra (33 frequency offsets; ±0.8 ppm; 0.05 ppm step size; and 0.05 µT RF saturation power) [32] and a B_1_ field map (flip angles of 30° and 60°, respectively) were obtained [26].

#### 2.2.3. Multi-Parametric MR Imaging

Multi-parametric MRI data were acquired and evaluated, including T1 and T2 relaxation maps, ADC maps, and CBF maps [42,43]. The data acquisition techniques were as follows:

(1) T1 Relaxation Maps:
  ∙ Pulse Sequence: RARE and variable time of recovery [RARE-VTR];
  ∙ Parameters: Six different TR values were employed (600, 900, 1500, 2500, 4000, and 7000 ms), with a constant TE of 12.2 ms, a rapid acquisition with the relaxation enhancement (RARE) factor set at 4, and a single NA.
(2) T2 Relaxation Maps:
  ∙ Pulse Sequence: Multi-spin multi-echo [MSME];
  ∙ Parameters: Fifteen TE values ranging from 10 to 150 ms in increments of 10 ms and a TR of 3000 ms, with NA set to 1.
(3) Apparent Diffusion Coefficient (ADC) Maps:
  ∙ Pulse Sequence: Single-shot spin-echo echo-planar imaging [EPI];
  ∙ Parameters: Seven diffusion weightings (b-values) were utilized, specifically set at 0, 166.7, 333.3, 500, 666.7, 833.3, and 1000 s/mm^2^, with a repetition time to echo time ratio (TR/TE) of 3000/18.7 ms, and NA was set to 3.
(4) Cerebral Blood Flow (CBF) Maps:
  ∙ Techniques: Flow-sensitive alternating inversion recovery [FAIR];
  ∙ Pulse Sequence: RARE with arterial spin labeling using variable inversion times [TI];
  ∙ Parameters: Sixteen inversion times (TIs) were applied, with TI_1_ set at 35 ms, TIs ranging from TI_2_ to TI_15_ spanning 100 to 1400 ms in 100 ms increments, and TI_16_ set at 1600 ms. The TR ranged from 10,212.2 to 11,777.2 ms, the TE was fixed at 36.36 ms, and a single NA was used.

#### 2.2.4. In Vivo ^1^H MR Spectroscopy

In vivo ^1^H MRS data were collected from a singular voxel (dimensions: 2.0 × 2.0 × 3.0 mm^3^; volume: 12 μL) located within the hippocampus. This was achieved through a spin-echo-based point-resolved spectroscopy sequence employing the variable power and optimized relaxation delay method (with the following parameters: TR/TE = 5000/16.3 ms, spectral width = 5 kHz, number of data points = 2048, and NA set at 256) [25,44].

### 2.3. Data Analysis

GluCEST images were examined, and the data analysis was performed using MATLAB (R2023a; Mathworks, Natick, MA, USA). To correct for B_0_ inhomogeneity, the Z-spectra derived from the WASSR data for individual voxels were subjected to fitting procedures. This process involves resetting the water signal within the chemical shift to 0 ppm, thereby extracting the frequency shift information for each voxel. Relative B_1_ values were determined by analyzing the B_1_ map. The computation of the GluCEST signal entailed the subtraction of the normalized magnetization signal at 3.0 ppm from the magnetization at the symmetrical reference frequency positioned upfield from water. GluCEST (%) = 100 × (M_[−3.0 ppm]_ − M_[+3.0 ppm]_)/M_[−3.0 ppm]_, where M_[−3.0 ppm]_ and M_[+3.0 ppm]_ are B_0_- and B_1_-corrected signals at ±3 ppm from water resonance, respectively [26]. Referring to the rat brain atlas [45], multi-slice T2-weighted images were obtained from all experimental animals. Subsequently, a single slice located between −4.0 mm and −4.4 mm posterior to the bregma along the anterior–posterior axis was chosen as the anatomical position for the quantification of the hippocampus. GluCEST signals across all rats were meticulously quantified using manually delineated regions of interest specifically drawn within the hippocampal region.

In vivo proton MR spectroscopy data quantification was performed through a fully automated pipeline, utilizing Linear Combination Model software (version 6.3-1D; copyright: Stephen W. Provencher, Stephen Provencher Inc., Oakville, ON, Canada) in conjunction with a set of simulated basis spectra. Metabolic concentrations (µmol/g) within the ^1^H MRS signal of the hippocampal region were quantified through water scaling and eddy current compensation, using the unsuppressed water signal as an internal reference for quantification. All the metabolite peaks were fitted within the chemical shift range of 4.3 to 0.3 ppm.

T_1_ and T_2_ relaxation maps were generated by applying the following equations: I(t) = I_0_ · [1 − C·exp(−TR/T_1_)] and I(t) = I_0_ · exp(−TE/T_2_). In the equation of the T_1_ relaxation map, C is a factor that considers incomplete inversion, where I(t) represent the signal intensity acquired at a specific TR and TE, and I_0_ denotes the equilibrium signal. The ADC map was generated through the application of the following equation: I = I_0_ · exp(−b·ADC). The CBF map was derived by reconstructing the images obtained with and without labeling.

### 2.4. Statistical Analysis

Statistical analyses were conducted using PASW Statistics 18 software (IBM Corp., Armonk, NY, USA). The GluCEST values and multi-parametric signals were separately quantified for the signals in the left and right hippocampal regions. Subsequently, the mean values for both sides were calculated, and a comparison of the mean values between the two groups was conducted. The detection of ^1^H MRS signals was conducted in the right hippocampal region, aiming to minimize interference from other brain tissues within the voxel. The GluCEST, ^1^H MRS, and multi-parametric imaging data were normally distributed (Kolmogorov–Smirnov test of normality, all *p* > 0.2). The signal intensities of GluCEST, ^1^H MRS, and multi-parametric images in the hippocampus of the FST and control groups were statistically analyzed using an independent *t*-test. Statistical significance was considered for *p* values < 0.05.

## 3. Results

**Figure 1:**
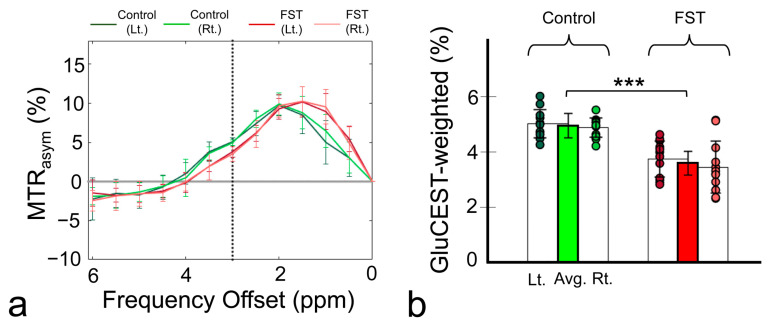
Magnetization transfer ratio asymmetry (MTR_asym_) spectra (**a**) and quantified glutamate-weighted chemical exchange saturation transfer (GluCEST)-weighted values (**b**) in the hippocampi of both study groups. The bar graphs depict the average GluCEST values in the left and right hippocampus, with the vertical lines on each bar indicating the corresponding standard deviations. The green and red bars indicate the averaged values between the left and right hippocampus in each group, respectively. Avg., average; FST, forced swimming test group; Lt., left; Rt., right. *** *p* < 0.001.

Figure 1 shows the magnetization transfer ratio asymmetry (MTR_asym_) curves (Figure 1a) and quantified GluCEST-weighted values (Figure 1b) in the hippocampus of the FST and control rats. The average GluCEST value of the left and right hippocampus was significantly lower in the FST group (3.67 ± 0.81%) than in the control group (5.02 ± 0.44%; *p* < 0.001).

**Figure 2:**
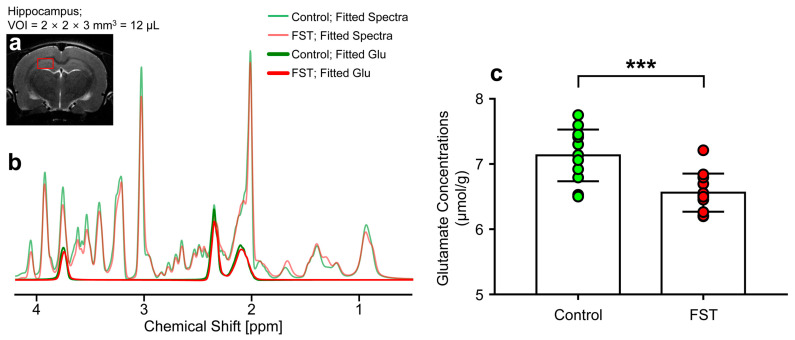
LCModel results of the proton spectra in the hippocampal region acquired from representative FST and control rats (**a**,**b**). The red rectangle represents the position of the volume of interest (**a**). (**c**) The bar chart with data points shows the mean glutamate concentration, while the vertical lines on each of the bars represent the standard deviations. FST, forced swimming test group; ppm, parts per million; VOI, volume of interest. *** *p* < 0.001.

Figure 2 illustrates the positioning of the voxel in the hippocampal region (Figure 2a) and the outcomes of spectral fitting for the ^1^H-MRS data in a representative rat from each group (Figure 2b). The quantified concentrations of Glu in the hippocampal region were notably reduced in the FST group compared to the control rats (Figure 2c) (6.560 ± 0.292 μmol/g vs. 7.133 ± 0.397 μmol/g, respectively; *p* = 0.001).

**Figure 3:**
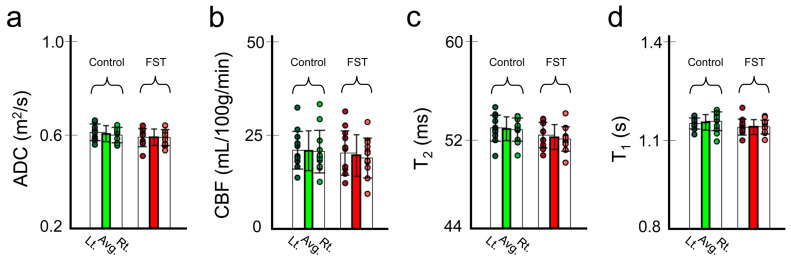
Calculated cerebral blood flow (CBF), apparent diffusion coefficient (ADC), T2, and T1 values in the left and right hippocampus (**a**–**d**). The green and red bars indicate the average values of both hippocampal regions in the control and FST groups, respectively. Avg., average; FST, forced swimming test group.

Figure 3 shows the evaluated multi-parametric MRI values (ADC, CBF, T_2_, and T_1_) in the hippocampal region in the FST and control group rats (Figure 3a–d). No significant intergroup differences were noted (all *p* ≥ 0.109). These results suggest that the multi-parametric values did not affect GluCEST signal formation.

**Figure 4:**
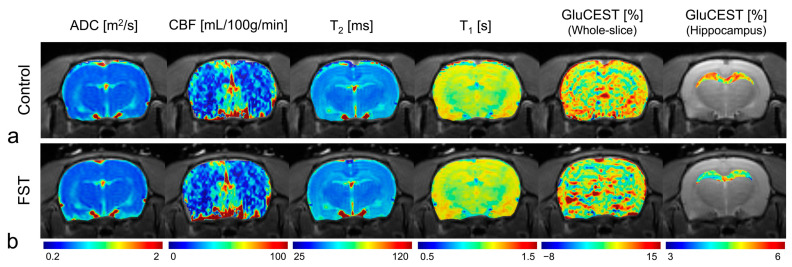
Reconstructed typical multi-parametric magnetic resonance images and GluCEST maps of the control (**a**) and FST (**b**) rats. ADC, apparent diffusion coefficient; CBF, cerebral blood flow; FST, forced swimming test group; GluCEST, glutamate-weighted chemical exchange saturation transfer.

Figure 4 displays reconstructed maps presenting quantified multi-parametric MRI and GluCEST values overlaid on the respective S0 images, as observed in a representative rat from both the FST and control groups (Figure 4a,b). While no noticeable distinctions were apparent in the visual inspections of the multi-parametric MRI (Figure 4), a notable contrast was observed in the GluCEST maps of the hippocampal region.

## 4. Discussion

Here, we employed a well-established modeling method, specifically, the FST, on rats to assess disorders associated with depression in the hippocampal region. In addition, to detect molecular-level changes in cerebral Glu, we conducted molecular MRI techniques using in vivo GluCEST and ^1^H MRS. The present study clearly showed that GluCEST signals and Glu concentrations were markedly lower in both hippocampal regions in FST versus control rats. Additionally, using multi-parametric MR imaging techniques, we evaluated various biological signals (T_1_, T_2_, ADC, and CBF) between FST rats and the control group; however, no statistically significant differences were observed between the two groups. Our findings demonstrate the capability to visualize specific areas of interest and quantitatively assess metabolic changes in various neuropsychiatric disorders.

Several studies to date focusing on depressive disorders have been conducted using animal models to report alterations in cerebral metabolite signals [10,23,46,47,48]. Li et al. [15] demonstrated significantly lower Glu levels in the hippocampal region of a depression-like rat model of chronic forced-swimming stress. Furthermore, Kumar et al. [14] observed a notable decrease in Glu levels in the hippocampi of rats using the chronic mild-stress technique, a depression-modeling method. Zhang et al. [16] used a chronic unpredictable mild-stress rat model that revealed significantly lower Glu/Cr ratios in both hippocampal regions in test subjects versus controls. In the study conducted by Akimoto et al. [17] using the high-resolution magic-angle spinning nuclear MRS technique, a significantly lower level of Glu in the hippocampal region of rats exposed to chronic stress was observed compared to control rats. These findings suggest the vulnerability of the Glu signaling system within the hippocampal region in depression-like disorders [21].

Glu, which functions as the principal excitatory neurotransmitter in the central nervous system (CNS), is released at synapses across the brain [49]. Moreover, Glu is a mediator of stress-induced neural alterations, with multiple preclinical findings suggesting its role as an additional mediator in the stress–depression relationship [14,42,50]. Additionally, numerous studies have reported that significantly reduced Glu levels in the hippocampus of patients with major depressive disorders may be due to hypercortisolism [5,51]. Along with Glu, cortisol is well known for its mediating role in stress-related disorders [52]. Moreover, hypercortisolism is associated with major depressive disorder, wherein cortisol interacts with glucocorticoid receptors in the hippocampus [53]. Lee et al. suggested that increased glucocorticoid levels escalate neurotoxicity, potentially causing neuronal loss and vulnerability in the susceptible hippocampal region [54]. Additionally, they highlighted that these outcomes may result in reduced hippocampal volume and neuronal damage, leading to the dysregulated metabolism of Glu, a pivotal neurotransmitter in the brain [54]. Thus, based on our study and previous research results, the significantly lower GluCEST-weighted levels and Glu concentrations observed in animal models exhibiting depression-like behaviors indicate the necessity for our future research to investigate neuropathological and biological studies related to neurotoxicity and glucocorticoid association. Furthermore, in future research, it is worthwhile to explore various depression-like animal models and modeling methods, evaluate metabolite substances at different timings (acute, subacute, and chronic), and investigate the therapeutic effects of antidepressants.

Although our research obtained results that could be utilized in various neuropsychiatric disorder domains, it has several limitations. First, it quantitatively evaluated cerebral Glu levels solely through a single FST in an animal model of depression-like behavior versus controls. It is necessary for future studies to observe cerebral metabolic changes induced by single versus chronic FST models as well as antidepressant treatments. Secondly, broadening the spatial scope of GluCEST imaging to identify signals within crucial regions linked to major depressive conditions necessitates the utilization of either whole-slice images or 3D GluCEST imaging techniques. Further research focusing on different brain regions prone to major depressive disorder, including both gray and white matter areas, is necessary. This research has the potential to provide understanding of the role of Glu in vivo in specific conditions and validate our discoveries. Finally, the present study has only revealed the fact regarding the changes in cerebral Glu observed in the depressive-like animal model. Hence, it is impossible to distinguish whether the cause of glutamate changes stems from depression, pain (chronic or acute), or another specific factor. To confirm and validate the observed changes in cerebral Glu in the FST model induced by depressive disorders, there is a need for a comprehensive approach involving various validation methods and studies encompassing biological, histological, and immunological research.

## 5. Conclusions

In summary, GluCEST imaging proved to be sufficiently sensitive at detecting and visualizing in vivo changes within the hippocampus region in a rat model of depressive-like behavior. Moreover, GluCEST promotes high-resolution functional imaging of cerebral Glu, offering the potential to provide metabolism-based diagnostic information distinct from conventional anatomically driven imaging diagnostic methods. The future implementation of this method could improve clinical outcomes of depressive disorders and other psychiatric conditions.

## Data Availability

The data that support the findings of this study are available from the corresponding author upon reasonable request.
